# Intermediate-risk thyroid carcinoma: indicators of a poor prognosis

**DOI:** 10.20945/2359-3997000000290

**Published:** 2020-08-24

**Authors:** Fernanda Nascimento Faro, Ângela Maria Leal Barros Bezerra, Nilza Maria Scalissi, Adriano Namo Cury, Marília Martins Marone, Carolina Ferraz, Rosália do Prado Padovani

**Affiliations:** 1 Irmandade da Santa Casa de Misericórdia de São Paulo Departamento de Medicina Divisão de Endocrinologia São Paulo SP Brasil Unidade de Doenças da Tireoide, Divisão de Endocrinologia, Departamento de Medicina, Irmandade da Santa Casa de Misericórdia de São Paulo, São Paulo, SP, Brasil; 2 Santa Casa de São Paulo Faculdade de Ciências Médicas São Paulo SP Brasil Faculdade de Ciências Médicas, Santa Casa de São Paulo, São Paulo, SP, Brasil; 3 Irmandade da Santa Casa de Misericórdia de São Paulo Serviço de Medicina Nuclear São Paulo SP Brasil Serviço de Medicina Nuclear, Irmandade da Santa Casa de Misericórdia de São Paulo, São Paulo, SP, Brasil

**Keywords:** Thyroid neoplasms, thyroid cancer, differentiated thyroid cancer, radioactive iodine therapy, thyroglobulin, prognosis

## Abstract

**Objective:**

The intermediate-risk (IR) category includes tumors with different degrees of aggression. We aimed to identify the risk factors associated with unfavorable response to initial treatment and compare the effect of low/high radioactive iodine (RAI) therapy.

**Subjects and methods:**

A total of 614 IR patients were selected from a database, during 1972-2015. All patients underwent total thyroidectomy and RAI therapy and were reclassified after 12-18 months into the favorable (complete/indeterminate) response group and the unfavorable (biochemical/incomplete structural) response group. A total of 92 patients were assessed for late response (mean: 9.19 ± 5.73 years). Age, gender, tumor size, histology, multifocality, vascular invasion, extrathyroidal extension, presence and number of lymph node metastasis, and stimulated thyroglobulin at ablation (sTg) were evaluated.

**Results:**

Mean age at diagnosis was 41.47 ± 15.81 years, and 83.6% of the patients were female. Within 12-18 months after initial therapy, unfavorable response was detected in 41.2% of the patients and was associated, in multivariate analysis, with lymph node metastasis (p = 0.041; odds ratio [OR] = 1.9), presence of more than five metastatic lymph nodes (p = 0,017; OR = 2.6), and sTg > 10 ng/mL (p = 0.005; OR = 10.0). For patients with a longer follow-up, sTg >10 ng/mL was associated with unfavorable response (p = 0.002; OR = 6.8). A higher RAI dose was not related to better prognosis at the end of the follow-up.

**Conclusion:**

A sTg level of >10 ng/mL and lymph node metastasis were associated with an unfavorable response 12-18 months after initial treatment. A RAI dose below 150 mCi was proven sufficient to treat IR patients.

## INTRODUCTION

The incidence of thyroid carcinoma, the most common endocrine cancer, has increased in recent decades ( 1 , 2 ). Although the condition of most patients with differentiated thyroid cancer (DTC) improves when properly treated, a proportion of them (14%-23%) experience disease recurrence or do not respond to conventional therapies ( 3 ). To improve the effectiveness of the treatment, the management of DTC should be individualized. For this purpose, the 2015 American Thyroid Association (ATA) guidelines suggest that patients should be staged after surgery to assess the risk of recurrence and persistence of the disease in addition to the risk of mortality ( 4 , 5 ).

Although initial staging is carried out, the recurrence and persistence rates within the same recurrence and persistence risk category may vary. For intermediate risk (IR), which is the second most common category of DTC (25%-35% of cases) ( 6 - 8 ), variations in the recurrence and persistence rates are relatively large (3%-30%) ( 5 ). This category includes a very heterogeneous population group. Within the same risk classification, it is possible to identify malignant tumors with a combination of different risk characteristics and, consequently, different degrees of aggressiveness.

Therefore, it remains unclear whether all patients classified in the IR category should receive the same initial treatment, especially radioactive iodine therapy (RAI) as adjunct therapy and perform the prescribed activity ( 5 , 6 , 9 - 11 ).

Considering the importance of differentiating patients with “low-risk characteristics” from those with “high-risk characteristics,” this study aimed to identify risk factors associated with tumor persistence/recurrence disease in a cohort of patients with intermediate risk of DTC recurrence. We also aimed to assess whether the use of a lower or a higher RAI dose (≤150 and >150 mCi) influences the outcome of these patients.

## SUBJECTS AND METHODS

### Patients and study design

A total of 614 IR patients were selected from the database of DTC of the Nuclear-Medicine Service, Nuclimagem, from 1972 to 2015. Nuclimagem is a nuclear medicine service that provides specialized care to patients in *Santa Casa de Misericórdia de São Paulo* . It receives patients not only from Santa Casa but also from all regions of the state, referred by the São Paulo State Health secretary to undergo treatments or examinations. That is why a long follow-up is not available for most of these patients.

All patients selected from the database fulfilled the ATA criteria for determine the IR of recurrence and had at least one of the following characteristics: i) aggressive histology (tall cell, columnar, insular, and solid variant), ii) T3 with microscopic invasion of the tumor into the perithyroidal soft tissues at initial surgery, iii) papillary thyroid cancer with vascular invasion, or iv) T-N1 (more than five lymph nodes involved whose sizes were between 0.2 and 3.0 cm without extranodal extension). Patients with positive anti-thyroglobulin antibodies (TgAb) were excluded from the study.

All patients underwent total thyroidectomy and RAI therapy with doses between 100 and 300 mCi after thyroid hormone withdrawal and were placed on a low-iodine diet in line with the ATA recommendations. A RAI dose of up to 150 mCi was considered as a low dose, while a dose above 150 mCi was considered as a high dose. After initial therapy, levothyroxine suppressive therapy was administered, as recommended by the ATA guidelines ( 5 ).

### Outcomes

Twelve to eighteen months after initial treatment (first assessment after ablation), all patients were reevaluated by measuring the levels of thyroid stimulating hormone (TSH)-stimulated thyroglobulin (Tg), and by performing a neck ultrasound (US), with or without a diagnostic whole-body scintigraphy (WBS). Clinical status was defined by classifying patients' response to initial treatment as excellent, indeterminate, biochemical incomplete, and structural incomplete, according to the 2015 ATA classification ( 5 ). Patients with excellent and indeterminate responses were classified as having a favorable response. On the contrary, biochemical or structural incomplete responses were classified as an unfavorable response.

Excellent response was considered if the imaging findings were negative and the patient had either a suppressed Tg level of <0.2 ng/mL or a stimulated Tg (sTg) level of <1.0 ng/mL. Indeterminate response indicated nonspecific findings on imaging studies, faint uptake in the thyroid bed on RAI scanning, and a nonstimulated Tg detectable level of ≥0.2 but <1.0 ng/mL or a sTg detectable level ≥ 1.0 but <10 ng/mL. Biochemical incomplete response was reported if the imaging findings were negative and the patient had either a suppressed Tg level of ≥1.0 ng/mL or a sTg level of ≥10 ng/mL. Structural incomplete response was indicated if there was presence of structural or functional evidence of disease with any Tg level.

Of the total patients selected from the database of the nuclear medicine service, 92 were examined in our endocrinology outpatient clinic ( *Irmandade da Santa Casa de Misericórdia de São Paulo* ). Therefore institution's, we were able to perform a long-term follow-up of these patients (mean: 9.19 ± 5.73 years) and reevaluate their response to the proposed treatment at the end of the follow-up.

### Study variables

The following risk factors were evaluated: age at diagnosis (<55 and ≥55 years), gender, tumor size (≤1 cm, 1-4 cm, and ≥4 cm at the largest diameter), histology (papillary or follicular carcinoma), histology of papillary carcinoma (classic, follicular, and others), histology of follicular carcinoma (minimally invasive or extensive invasive), multifocality, vascular invasion, extrathyroidal extension, presence and number of lymph node metastasis (≤5 and >5 metastatic lymph nodes), RAI doses (≤150 mCi and >150 mCi), and sTg at ablation (<5 ng/mL, 5-10 ng/mL, and >10 ng/mL).

### Assays

All patients included in our study underwent the first RAI treatment until 2015.

The analyses of sTg at ablation and TgAb were performed in the same laboratory and the levels were assessed using the immulite thyroglobulin assay. This is a first-generation Tg assay with a functional sensitivity (FS) of 1.0 ng/ml and a lower limit of detection of 0.2 ng/mL.

For patients who were assessed for response to initial treatment until 2015, sTg was used because, until then, second-generation Tg assays were not available in our laboratory.

After 2015, patients' response to initial treatment was evaluated by measuring the level of suppressed or sTg because, at that time, second-generation Tg assays started to become available in our institution. Hence, thereafter, Tg levels were assessed using the chemiluminescent assay (Access Thyroglobulin Assay; Beckman Coulter) with an FS of 0.1 ng/mL.

### Imaging methods

Neck US was performed by experienced ultrasonographers from our institution using a high-resolution color Doppler US apparatus with a 7.5-MHz linear transducer. Diagnostic or post-therapeutic 131I-WBS was performed using a one-head – camera (Apex SPX 4000; Elscint Italia, Milan, Italy) with a high-energy collimator and with a sensitivity of 160 cpm/mCi. The scan speed was 10 cm/min with total counts of at least 100,000 cpm.

### Statistical analysis

Statistical analysis was performed using SPSS version 20.0. Categorical variables were expressed as absolute and relative frequencies and were compared using chi-square or Fisher's exact test when appropriate. Continuous variables were expressed as mean and standard deviation.

Univariate analysis and multivariate logistic regression models were used to investigate the association between unfavorable response and clinical variables. A multivariate logistic regression model was used to identify independent prognostic factors of recurrence. Odds ratios (ORs) were calculated for the variables that were significant in the univariate and multivariate analyses. A p value of <0.05 was considered to indicate statistical significance.

To evaluate the effect of RAI activities on prognosis, we analyzed the association between RAI doses (≤150 and >150 mCi) and an unfavorable response at the end of the follow-up. This association was exclusively evaluated in the subgroup of patients with an initial unfavorable response, considering the hypothesis that this particular group would more likely benefit from higher doses of RAI.

The study was approved by the local research ethics committee.

## RESULTS

A total of 614 patients diagnosed with intermediate-risk DTC according to the 2015 ATA risk stratification guidelines were studied. Mean age at diagnosis was 41.47 ± 15.81 years. Approximately 83.6% of the total patients were female, and 86.8% had papillary thyroid carcinoma. Moreover, 44.8% of the patients had lymph node metastasis. The serum Tg value at ablation was less than 5 ng/mL in 87.9% of the patients, and 61% received high RAI therapy (>150 mCi). When patients were reevaluated 12-18 months after the initial therapy, 58.8% achieved a favorable response (complete or indeterminate), while 41.2% showed unfavorable response (biochemical or structural incomplete) ( Table 1 ). The frequency of incomplete structural response was higher in males, when compared to incomplete biochemical response (p = 0.031; OR = 2.08 [95% confidence interval (CI): 1.06-4.04]).

**Table 1:** Baseline characteristics of 614 intermediate-risk DTC patients according to the American Thyroid Association risk stratification

Characteristics		%	n
Age (years) at diagnosis			
	<55	77.2%	474
	≥55	22.8%	140
Gender			
	Female	83.6%	513
	Male	16.4%	101
Histology			
	Papillary	86.8%	533
	Follicular	13.2%	81
Histology variant of papillary carcinoma			
	Classic	65.1%	343
	Folicular	27.9%	147
	Others	7.0%	37
Histology variant of follicular carcinoma			
	Minimally invasive	93.5%	72
	Extensively invasive	6.5%	5
Tumor size (cm)			
	≤1	18.3%	97
	>1-4	53.1%	281
	≥4	28.5%	151
	Multifocality	30.9%	190
	Extrathyroidal invasion	17.9%	110
	Vascular invasion	37.4%	201
	Presence of metastatic lymph nodes	44.8%	275
Number of metastatic lymph nodes			
	≤5	81.2%	437
	>5	18.8%	101
	Stimulated Tg at ablation (ng/mL)		
	<5	87.9%	333
	5-10	3.7%	14
	>10	8.4%	32
RAI dose (mCi)			
	≤150	39.0%	225
	>150	61.0%	352
Response classification to initial therapy			
	Complete	29.2%	179
	Indeterminate	29.6%	182
	Biochemical incomplete	27.2%	167
	Structural incomplete	14.0%	86

n = 614; RAI: radioactive iodine.

In the univariate analysis, the risk factors associated with unfavorable response 12-18 months after initial therapy were male gender (p = 0.022), tumor size >4 cm (p = 0.038), presence of lymph node metastasis (p < 0.001), more than five lymph node metastasis (p < 0.001), and serum sTg value at ablation higher than 10 ng/mL (p < 0.001) ( Table 2 ). Multivariate analysis confirmed the association between unfavorable response and lymph node metastasis, number of lymph node metastasis, and sTg value at ablation ( Table 3 ).

**Table 2:** Univariate analysis of factors associated with incomplete response 12–18 months after initial therapy and the OR of significant factors

Characteristics		Complete/Indeterminate	Incomplete	P value	Test	OR *	95% CI for OR	
							Lower	Upper
Age (years) at diagnosis	<55	282	192	0.517	Chi-square			
	≥55	79	61					
Gender	Female	312	201	0.022	Chi-square	1 *		
	Male	49	52			1.65	1.07	2.53
Histology	Papillary	307	226	0.122	Chi-square			
	Follicular	54	27					
Histology variant of papillary carcinoma	Classic	191	152	0.312	Chi-square			
	Folicular	88	59					
	Others	25	12					
Histology variant of follicular carcinoma	Minimally invasive	49	23	0.329	Fisher's Exact			
	Extensively invasive	2	3					
Tumor size (cm)	≤1	69	28	0.038	Chi-square	1 *		
	>1-4	169	112			1.63	0.99	2.69
	≥4	83	68			2.02	1.17	3.48
Multifocality	No	253	171	0.511	Chi-square			
	Yes	108	82					
Extrathyroidal invasion	No	293	211	0.477	Chi-square			
	Yes	68	42					
Vascular invasion	No	187	150	0.128	Chi-square			
	Yes	125	76					
Presence of metastatic lymph nodes	No	227	112	0.000	Chi-square	1 *		
	Yes	134	141			2.13	1.54	2.96
Number of metastatic lymph nodes	≤5	272	165	0.000	Chi-square	1 *		
	>5	40	61			2.51	1.61	3.92
Stimulated Tg at ablation (ng/mL)	<5	214	119	0.000	Chi-square	1 *		
	5-10	13	1			0.14	0.02	1.07
	>10	7	25			6.42	2.70	15.29

* OR reference: category 1; OR: odds ratio; CI: confidence interval; Tg: serum thyroglobulin; RAI: radioactive iodine.

Significant difference at p ≤ 0.05.

**Table 3:** Factors associated with incomplete response by multivariate analysis

Characteristics		OR	95% CI for OR		P value
			Lower	Upper	
Presence of metastatic lymph nodes	Yes	1.905	1.028	3.528	0.041
Number of metastatic lymph nodes	>5	2.634	1.187	5.846	0.017
Stimulated Tg at ablation (ng/mL)	>10	10.095	2.044	49.863	0.005

* OR: odds ratio; CI: confidence interval; Tg: serum thyroglobulin; RAI: radioactive iodine.

Significant difference at p ≤ 0.05.

All factors that were significant in the univariate analysis were included in the multivariate analysis ( Table 2 ): gender, tumor size, stimulated Tg at ablation, and presence and number of metastatic lymph nodes.

Of the 92 patients who were evaluated after a long-term follow-up (mean: 9.19 ± 5.73 years), 65.2% showed a favorable response 12-18 months after initial therapy ( Table 4 ). The majority of the patients maintained a favorable response, and only 3.3% had favorable to unfavorable response. Of the patients initially classified as having an unfavorable response, 31.3% showed a favorable response at the end of the follow-up ( Table 4 ).

**Table 4:** Evaluation of 92 intermediate-risk DTC patients according to response range 12-18 months after initial therapy and after a long-term follow-up (mean; 9.19 ± 5.73 years)

Response range after 12-18 months of initial therapy	Response range at the end of follow-up		P value
	Favorable	Unfavorable	
Favorable	58	2	0,000
Unfavorable	10	22	

* Favorable: complete or indeterminate response. Unfavorable: biochemical/structural incomplete response.

Significant difference at p ≤ 0.05.

A total of 14.1% of the patients developed distant metastasis, 16.4% underwent another surgery, and 19.5% received another dose of RAI. We also observed that patients who achieved an excellent response to therapy in the first 2 years of evaluation had a substantially lower risk of recurrence/persistence than those who presented an incomplete response to therapy. Moreover, 93% of patients who had a complete response in the initial stratification had the same response at the end of the follow-up. Regarding patients who had an incomplete response at the initial assessment, 58% achieved the same response in the final evaluation ( Table 5 ).

**Table 5:** Initial and final response of 92 patients who had a longer follow-up

Initial Response Type	Final Response Type				Total
	Complete	Indeterminate	Biochemical incomplete	Structural incomplete	
Complete	**93%**	3%	0%	5%	100%
Indeterminate	60%	**40%**	0%	0%	100%
Biochemical incomplete	23%	8%	**54%**	15%	100%
Structural incomplete	21%	11%	11%	**58%**	100%
Total	61%	13%	10%	16%	**100%**

The characteristic associated with an unfavorable response at the end of the follow-up in the univariate (p = 0,002) and multivariate (p = 0.002; OR = 6.87 [95% CI: 2.03-23.2]) analyses was sTg at ablation higher than 10 ng/mL ( Table 6 ).

**Table 6:** Factors associated with incomplete response after a long-term follow-up (mean: 9.19 ± 5.73 years)

Characteristics		Favorable	Unfavorable	P value	Univariate analysis			P value	Multivariate analysis		
					OR	95% CI for OR			OR	95% CI for OR	
						Lower	Upper			Lower	Upper
sTg at ablation (ng/mL)	<5	38	7	0.002	1 *						
	5-10	6	2		1.81	0.30	10.86				
	>10	8	12		8.14	2.44	27.15	0.002	6.875	2.030	23.283
Constant								0,000	0,200		

* OR reference: category 1; OR: odds ratio; CI: confidence interval; sTg: stimulated serum thyroglobulin.

Significant difference at p ≤ 0.05.

With regard to the effect of high and low RAI doses on patients' prognosis, no significant association was observed between RAI doses and response range at the end of the follow-up (p = 0.699), demonstrating that using RAI doses higher than 150 mCi had no effect on long-term outcomes ( Table 7 ).

**Table 7:** RAI doses and response range at the end of the follow-up

Characteristics		Favorable	Unfavorable	P value
RAI dose (mCi)	≤150	5	8	0,699
	>150	5	14	

* RAI: radioactive iodine.

Significant difference at p ≤ 0.05.

## DISCUSSION

The IR category proposed by the 2015 ATA guideline is very heterogeneous and comprises tumors of different degrees of aggressiveness. Low-risk and high-risk patients should be provided with individualized treatment and should not receive insufficient or unnecessary therapies. In addition, recognizing high-risk patients is of utmost importance to recommend a more aggressive approach, and these patients should be closely followed up to determine who among them will benefit from the treatment.

In this sense, the 2015 ATA guideline recommends the selective use of RAI for IR patients. In fact, RAI treatment should be considered for patients of this category but who also have a higher risk of persistent or recurrent disease. However, which characteristics represent an important risk of recurrence or persistence and who among the IR patients deserve a more aggressive treatment remain controversial ( 5 ). So, the main objective of this study was to identify risk factors associated with tumor persistence/recurrence disease in a cohort of patients with intermediate risk of DTC recurrence.

First, it is important to highlight some of the characteristics of this study. The present study used a large sample size and determined the patients' response to initial therapy at the first 12-18 months after RAI; we also evaluated the long-term (mean: 9.19 ± 5.73 years) results of a significant number of patients. Furthermore, all patients initially underwent the same surgical treatment and received RAI treatment in the same institution using the same protocol.

Our study demonstrated, using a large cohort of IR patients at risk for recurrence and persistent disease, that the risk factors associated with unfavorable response to initial therapy were male gender, larger tumor size (>4 cm), presence of lymph node metastasis, and a sTg value at ablation higher than 10 ng/mL. Lymph node metastasis, number of metastatic lymph nodes, and level of sTg at ablation were considered independent risk factors for incomplete response 12-18 months after the initial treatment; moreover, sTg was associated with incomplete response in a longer follow-up.

Bandeira and cols. ( 12 ), Bernier and cols. ( 13 ), and Ballal and cols. ( 7 ) showed the prognostic value of sTg measured before RAI therapy and suggested that a higher sTg value at this point, the greater the likelihood of an incomplete response to initial treatment. Bandeira and cols. ( 12 ) proposed that 3.75 ng/mL is a good cut-off value for incomplete response. Meanwhile, Ballal and cols. ( 7 ) demonstrated that a sTg level at first follow-up higher than 10 ng/mL was a risk factor for a significant reduction in event-free survival. Our findings corroborate with the results of these studies and emphasized the importance of checking the sTg value after surgery to help clinicians decide the appropriate RAI therapy. Inappropriately elevated postoperative Tg values are being used as a basis to recommend the appropriate RAI adjuvant therapy, even in the absence of structural disease ( 7 , 12 - 18 ).

The ATA guideline also recommends RAI adjuvant therapy in older patients with large primary tumors (>45 years, >4 cm) because of the significant risk of recurrence or distant metastasis ( 5 ). Tuttle and Sabra and Ballal and cols. ( 7 , 9 ) demonstrated that these variables were associated with an incomplete response and that patients with these characteristics can benefit from adjuvant treatment with RAI (100-150 mCi). Our results were in line with their data regarding tumor size bigger than 4 cm but not regarding the association of age worst prognosis.

The relationship between DTC recurrences and gender was evaluated in some studies. Only one previous study showed less disease recurrence in female patients, with another study noting less recurrence in this group after conducting only univariate analysis. Although most of these studies showed some positive effect of female gender on overall survival and disease-specific survival (DSS), a female survival advantage is not a universal finding, and the effect of gender on recurrences of DTC was not substantiated ( 19 ). In our study, male gender was a risk factor for an unfavorable response; however, when adjusted for other variables, it was not significant, showing that the influence of gender on prognosis depends on other characteristics.

Compared with that of incomplete biochemical response, the frequency of incomplete structural response was more prevalent in males than in females. This results corroborate with Nilubol and cols.'s findings ( 20 ), which showed that men with thyroid cancer are more likely to have more advanced and aggressive cancer, although this study did not conclude that male gender was an independent predictor of DSS.

In accordance with the findings reported in literature, vascular invasion increases the risk of distant metastases. Hence, RAI therapy should be considered in these patients to facilitate initial staging and follow-up ( 21 ). In the present study, vascular invasion was not associated with an unfavorable response, which is inconsistent with the findings of previous publication.

Regarding lymph node involvement, the current guidelines do not recommend the use of RAI therapy for all patients with lymph node metastases ( 3 , 5 ). Tuttle and Sabra ( 9 ) and Ballal and cols. ( 7 ) proposed the selective use of RAI only in high-risk patients with N1 disease (more than five involved lymph nodes), considering that these patients carry a risk of recurrence (>20%). Consistent with these findings, our study also demonstrated that the presence of lymph node involvement in IR patients should be considered as an independent risk factor for incomplete response ( 5 , 7 ), especially in the presence of more than five metastatic lymph nodes, and an indication for adjuvant RAI therapy.

A differential of this study was the comparison between the effect of different RAI doses in the IR patients' response to the initial treatment. To the best of our knowledge, only a few studies in literature compared the effect of different RAI doses in IR patients with different risk characteristics. Castagna and cols. ( 6 ) was one of the authors who carried out a study to compare the effect of RAI doses below and above 100 mCi (3,700 MBq) in the success of the ablation and in the outcome of IR patients. Similar to our results, no significant difference was observed in terms of the effect between these activities. Our data also showed no benefit related to the response to initial treatment in patients treated with high dose of RAI (>150 mCi). We analyzed the response after 12-18 months and after a mean of 9 years. In the subgroup initially classified as having an incomplete response, no significant difference was observed between lower or higher doses of RAI.

Moreover, our study reported that 96.7% of the patients initially classified as having a favorable response and 58% patients initially classified as having an incomplete response maintained this status after 9 years of follow-up. These findings are consistent with the results reported in literature ( 22 ).

Unfortunately, a long-term follow-up of these patients was not performed, and we consider this as a limitation of the study. However, the assessment of 92 patients who have been followed up for a longer period confirmed that a risk-adapted approach to follow-up cannot be based solely on static, initial estimates of risk, as previously suggested by other authors ( 5 , 11 ). Vaisman and cols. clearly showed the importance of the restratification in several cohorts worldwide and in Brazil ( 22 ).

The other limitations of the present study are follows: although RAI was administered in the same laboratory, using the same protocols and type of preparation, surgery and follow-up of patients were not carried out at the same center. The Nuclear Medicine Division of *Santa Casa de São Paulo* , as a referral center, receives patients from several other centers in the region to undergo RAI therapy. Consequently, variables such as surgical ability, extent of surgery (with or without lymphadenectomy), and follow-up protocols specific to each service may be considered as confounding factors ( 6 , 12 ).

In conclusion, our study provides further evidence that some risk factors are associated with worst prognosis in IR DTC patients, specifically the presence of lymph node metastasis, number of involved nodes, and high serum Tg levels at ablation, which increase the risk of an unfavorable response. For IR patients, sTg ablation should be evaluated and values above 10 ng/mL should be considered an indication for a more aggressive treatment and a closer follow-up. In the context of adjuvant therapy, doses lower or equal to 150 mCi appear to be sufficient for treating IR patients.
